# Influence of Education Level of Older Patients on Polypharmacy, Potentially Inappropriate Medications Listed in Beer’s Criteria, and Unplanned Hospitalization: A Cross-Sectional Study in Lahore, Pakistan

**DOI:** 10.3390/medicina54040057

**Published:** 2018-08-24

**Authors:** Muhammad Rehan Sarwar, Sadia Iftikhar, Muhammad Sarfraz

**Affiliations:** 1 Department of Pharmacy, The Islamia University of Bahawalpur, Punjab 63100, Pakistan; 2 Department of Pharmacy Practice, Akhtar Saeed College of Pharmaceutical Sciences, Lahore 54000, Pakistan; thesadiyaiftikhar@gmail.com; 3 Department of Pharmaceutics, College of Pharmacy, Al Ain University of Science and Technology, Al Ain, PO Box 64141, Abu Dhabi, UAE; muhammad.sarfraz@aau.ac.ae

**Keywords:** Beer’s criteria, older people, potentially inappropriate medicines, education level, unplanned hospitalization, polypharmacy

## Abstract

*Objective*: To evaluate influence of education level of older patients on polypharmacy, potentially inappropriate medications (PIMs) listed in Beer’s Criteria, and unplanned hospitalization. *Methods*: A cross-sectional study was conducted among older people aged ≥65 years between 1 December 2017 and 28 February 2018. For data analysis, descriptive statistics and logistic regression analysis were employed. *Results*: Among 385 older patients, 88.8% were prescribed PIMs and 56.4% underwent PIMs associated unplanned hospitalization. Older people were less exposed to polypharmacy or excessive polypharmacy as their education levels increased (no formal education vs. primary vs. secondary vs. tertiary, 74% vs. 69.8% vs. 60.5% vs. 58.1%). Patients having higher education were also accompanied by significantly lower prescription of PIMs (no formal education vs. primary vs. secondary vs. tertiary, 96% vs. 87.3% vs. 84.5% vs. 79.1%) as well as unplanned hospitalization (no formal education vs. primary vs. secondary vs. tertiary, 64.7% vs. 76.2% vs. 40.3% vs. 46.5%). Results of regression analysis revealed that no formal education (OR = 1.202, 95% CI = 1.032–2.146, *p*-value = 0.003) and primary education level (OR = 1.175, 95% CI = 1.014–1.538, *p*-value = 0.039) were significantly associated with the use of polypharmacy among older people. On the other hand, no formal education was significantly associated with the prescription of PIMs (OR = 1.898, 95% CI = 1.151–2.786, *p*-value = 0.007). Furthermore, older people with no formal education (OR = 1.402, 95% CI = 1.123–1.994, *p*-value = 0.010) and primary education level (OR = 1.775, 95% CI = 1.281–3.018, *p*-value = <0.001) were significantly more likely to undergo unplanned hospitalization. *Conclusions:* Patients having low literacy level are more likely to receive PIMs, polypharmacy, and undergo unplanned hospitalization in comparison to highly educated patients. Hence, promotion of health literacy for patients is crucial to overcome these problems.

## 1. Introduction

Worldwide, the over-utilization of healthcare resources by the older population is a result of the increase in number of patients belongs to this age group [1,2]. The prime focus of pharmacotherapy given to elderly patients is to improve their quality of life (QoL). The older patients are at high risk of developing adverse drug events (ADEs) due to various factors (e.g., polypharmacy, comorbidities and age-related physiological changes) that may negatively affect the pharmacokinetics and pharmacodynamics properties of therapeutic agents [3,4,5,6,7,8,9]. The chances of unexpected admission of older people to the inpatient departments of healthcare settings because of medicine associated adverse health outcomes become several folds higher that cannot be managed in outpatient settings and it is termed as “unplanned hospitalization” [10].

The prescribing practices of potentially inappropriate medications (PIMs), especially when better and effective drugs are available, refer to the use of such medicines whose risk may outweigh the beneficial therapeutic outcomes. The prescribing of these medications can be termed as potentially inappropriate prescribing (PIP) [11]. The utilization of PIMs by the older patients is common globally. Studies conducted in India and New Zealand revealed that nearly 23.6% and 42.7% of the older patients were prescribed with PIMs, respectively [12,13]. Such practices are associated with the development of ADEs and unplanned hospitalization [14]. Evidence suggested that each year nearly 1 million older people are hospitalized due to the untoward effects of PIMs in the United States of America (USA) [15,16]. Similar estimates have been made in Australia where almost 15–22% of the senior citizens are annually hospitalized due to over prescribing of PIMs [17]. The consumption of inappropriate drugs compromises patient’s safety, negatively affects the quality of life (QoL) and dramatically increases the rate of mortality and morbidity, so there must be limited use of these hazardous medicines [18,19,20,21,22,23]. For this reason, the American Geriatric Society (AGS) has developed guidelines which are named as Beers Criteria for Potentially Inappropriate Medication Use in Older Adults [8,24,25,26]. Other criterion like Screening Tool of Older Persons’ potentially inappropriate Prescriptions (STOPP) and the Screening Tool to Alert doctors to Right Treatment (START) were developed in Ireland [27]. Factors like polypharmacy and education level of patients are primarily responsible for this problem. The term “polypharmacy” means the over consumption of a single drug and the utilization of multiple therapeutic agents [28]. This factor makes the treatment regimen complex and is also responsible for poor patient compliance towards the prescribed therapy [29,30]. Evidence revealed that there is a strong relationship between the education level and health status of the patients. This might be attributable to the reason that highly educated individuals are more likely to understand their health needs and communicate more effectively with the healthcare professionals [31].

Older population is growing at a much faster rate. The census conducted between 1990 to 2010 in Pakistan revealed that older population has increased by 75.1% [32]. In 1998, it was also reported by the World Health Organization (WHO) that the population aged ≥65 years represent 5.6% of the entire population of Pakistan with a probability of increasing to 11% by the year 2025 [33]. Increase patient load has affected the efficient working of the public hospitals. This has caused an acute shortage of physicians, pharmacists, nurses and paramedical staff [34]. The outpatient departments (OPDs) of public hospitals are mostly run by medical officers and fresh medical graduates while the senior consultants visit OPDs for the specific and short duration of time. At a time, two or more patients have to share one bed in the hospital. In developed countries, one physician examine five to seven patients in a day but in the OPDs of public hospitals of Pakistan each physician has to examine approximately 100 patients daily [35]. Insufficient geriatric health services have raised the risk of increased mortality rate of older patients and it ultimately badly affects the healthcare system of Pakistan in the region of South Asia. Low state spending on the health of older people, abject poverty and low literacy rate of patients has indirectly worsened the healthcare system in Pakistan [36]. In the developing world, demographic rises in the number of older people and the availability of scarce literature on PIMs indicate a need for studies in this area. These studies will provide a landmark for stakeholders in making policies, determining the impact of medicines on community level and prioritizing the medical needs. The previously published studies conducted in different regions of the world have shown the effect of using Beers criteria as a guide demonstrated an increasing trend in the use of PIMS. However, it is still ambiguous that whether the utilization of PIMs listed in Beers criteria can lead to the development of ADEs in the older people or not. However in Pakistan, no data have been made available on zonal, provincial and national level that gives an insight about the consumption of PIMs by the older patients. Till date no clear estimates have been made about PIMs associated unplanned hospitalization of older adults. Thus, the present study aims to evaluate influence of education level of older patients on polypharmacy, PIMs listed in Beer’s Criteria, and unplanned hospitalization.

## 2. Materials and Methods

### 2.1. Study Design

A quantitative, observational, cross-sectional and prospective study was conducted to scrutinize the influence of education level of older patients on polypharmacy, PIMs listed in Beer’s Criteria, and unplanned hospitalization.

### 2.2. Study Settings

For this study, we divided Lahore into four zones i.e., north, south, east and west and obtained data from four different tertiary care hospitals of Lahore, Punjab, Pakistan (1: Bahria international hospital, 2: Doctors hospital, 3: Mayo hospital, and 4: Surgimed hospital). The healthcare settings were randomly selected and data were collected between 1 December 2017 and 28 February 2018. These selected settings were tertiary care public and private hospitals where inpatient healthcare services were also provided to the patients’ aged ≥65 years. These were comparable in terms of staff, services and availability of formulary medicines, thus healthcare professionals followed the same prescribing practices. The random selection of patients from these tertiary care hospitals could have minimized the chances of selection bias.

### 2.3. Study Population and Sample Size

According to latest Pakistani census, the total population living in Pakistan is 201,995,540. Lahore is the capital city of Punjab province and the second largest city of Pakistan with an estimated population of 11,126,285 people [37]. By using Raosoft sample size calculator [38], minimum obligatory sample size was 385 with 95% confidence interval (CI) and 5% margin of error (Equation (1)).

n = Nx/((N − 1) E2 + x)(1)

where n = population size, x = CI, E = margin of error. Records of those patients were included in the study that were ≥65 years of age, chronically ill and hospitalized. While, the records of all those older patients were excluded who were hospitalized on planned basis for acute illness (burn, appendicitis), exacerbation of chronic diseases or infections (Alzheimer’s disease, osteoporosis), end-stage life threatening diseases (cancer, HIV/AIDS), providing pre and post-surgery medical care, palliative care and short-term prognosis, had incomplete medical records.

### 2.4. Data Collection

A data collection form was designed that consisted of five major parts: (1) demographic characteristics, (2) socio-economic characteristics, (3) health-related characteristics, (4) clinical indications, and (5) past medication history. SPSS version 21.0 was used for calculation of reliability coefficients. Internal consistency was measured by Cronbach’s alpha, while reproducibility was evaluated by using intra-class correlation for each item in the scales, with acceptable values ≥0.6. The Cronbach’s alpha value was 0.74 demonstrating the excellent reliability. A pilot study was undertaken between October and November, 2017 for pre-testing the study instrument.

### 2.5. Measurements

#### 2.5.1. Demographic Characteristics

The following characteristics were evaluated in the demographic data of selected patients; gender (male/female), age (65–74, 75–84 and ≥84) and civil status (single, married, widowed, and divorced).

#### 2.5.2. Socioeconomic Characteristics

The socioeconomic characteristics were; education level (no formal education, primary, secondary, tertiary), employment status (employed, unemployed, retired), annual income (low class, middle class, upper class), residence (rural, urban). The patients who were unemployed but received revenue from their lands, business, or pensions were considered as employed.

#### 2.5.3. Health-Related Characteristics

Prescriptions were used to collect medical data while patients’ attendants were consulted for demographic, socio-economic and health-related information. Health-related characteristics included the following parameters: Self-reported health (good, moderate, poor), health service utilization (number of clinic visits of ≤3/year and ≥4/year), health risks (smoking, alcohol consumption, obesity) and comorbidities (present, absent) which included chronic diseases like diabetes mellitus, coronary vascular disease, respiratory, gastrointestinal, joint diseases, hypertension, central nervous system disorders, etc. Body mass index (BMI) was used to determine obesity and patients were considered as either normal (BMI < 25 kg/m^2^), over weight (25 ≤ BMI < 30 kg/m^2^) or obese (BMI ≥ 30 kg/m^2^) [39].

#### 2.5.4. Drug Utilization Evaluation

After collecting data from all the patients, a list of PIMs was made for categorizing the drugs as low, medium and highly prescribed medications. The Anatomical Therapeutic Chemical Classification system (ATC) was used for the estimation of drug utilization patterns [40]. The prescribed active substances were classified as: Low (prescribed to <10% of selected patients), medium (prescribed to ≥10% of the selected patients but <40%) and high (prescribed to >40% of selected patients).

#### 2.5.5. Potentially Inappropriate Medications Evaluation

For examining PIMs, medicines prescribed to the selected patients were evaluated according to the 2015 Beer’s Criteria [41]. Detection of PIMs was based on past medication history of patients. All the drugs mentioned in the past medication history were checked for appropriateness with respect to indications and interactions. Disease dependent PIMs were defined on the basis of International Classification of Diseases, Ninth Revision, Clinical Modification (ICD-9-CM) codes [42]. The expert opinions of physicians and clinical pharmacists were also taken in account before reaching the final decision.

#### 2.5.6. Unplanned Hospitalization Evaluation

All those selected cases were referred to as “unplanned hospitalization” in which patients visited the emergency department and their clinical sign and symptoms did not improve within 24 h, and there had been the need of further investigation or treatment which compelled the healthcare professionals to admit them in the inpatient ward directly from the emergency room. Therefore, the patient’s profile of all the selected participants were scrutinized to check the clinical presentation and diagnostic tests results at the time of their visit to the emergency room as well as unplanned admission in the inpatient ward. On the basis of diagnostic findings and the sound clinical judgment of physicians and clinical pharmacist, if the possibility of unplanned hospitalization due to underlying diseases was ruled out then the patients were asked to show their all prescriptions and pertinent lab test results as well as diagnostic findings one month prior to their hospitalization. According to the guidelines of American Academy of Family Physicians (AAFP), it is rare for any drug that the interval between initiation of therapy and onset of adverse drug reaction is less than 1 week or more than 1 month [43]. Thus, the utilization of PIMs one month prior to unplanned hospitalization was evaluated. All the information pertaining to drug utilization before unplanned hospitalization was then confirmed by conducting face-to-face interviews of the patient’s attendants. Based on past medical history, any such drug was said to be PIM if it was contraindicated to the patient according to Beer’s Criteria [41]. In this way the utilization of any PIM by the patients within 1 month prior to their unplanned hospitalization was checked.

### 2.6. Statistical Analysis

Statistical Package for Social Sciences (IBM Corp. Released 2012. IBM SPSS Statistics for Windows Version 21.0. IBM Corp., Armonk, NY, USA) and Microsoft Excel (MS Office, 2010. Microsoft Corp., WA, USA) were used for data analysis. Descriptive statistics such as frequencies and percentages were used to present the data. Furthermore, logistic regression analysis was performed to evaluate the influence of educational level on polypharmacy, PIMs and unplanned hospitalization. Results were expressed as Odds Ratio (OR) accompanied by 95% Confidence Intervals (95% CI) and a *p*-value < 0.05 was used for statistical significance of differences.

### 2.7. Ethics Approval and Consent to Participate

Ethical Approval including verbal informed consent process was obtained from Pharmacy Research Ethics Committee of Akhtar Saeed College of Pharmaceutical Sciences, Lahore (Reference: 08-2017/PREC, 22 September 2017). Before conducting the study, permission was granted from the hospital administrators. The purpose and protocols of this study were thoroughly explained to every participant and their verbal consents were obtained. Written consent was not possible for most of the respondents because either they acquired no formal education or they had problems in reading and/or signing the consent document.

## 3. Results

### 3.1. Characteristics of the Participants

A total of 419 older hospitalized patients in government and private hospitals of Lahore were approached and 385 consented patients (response rate = 91.8%) were included according to inclusion and exclusion criteria.

Nearly two-thirds (66.2%, *n* = 255) of the participants were male and 68.1% (*n* = 262) were 65–74 years of age. More than one third (39.0%, *n* = 150) of them acquired no formal education and 31.2% (*n* = 120) were from low annual income class. Self-reported health was moderate in 60.0% (*n* = 231) whereas 55.3% (*n* = 213) had ≥3 clinic visits in the previous year. Two-thirds (67%, *n* = 258) were subjected to polypharmacy (five to nine drugs) or excessive polypharmacy (utilization of ten or more drugs) while 88.8% were prescribed with PIMs and just over one half (56.4%, *n* = 217) underwent PIMs associated unplanned hospitalization. Table 1 showed that older people were less exposed to polypharmacy or excessive polypharmacy as their education levels increased (no formal education vs. primary vs. secondary vs. tertiary, 74% vs. 69.8% vs. 60.5% vs. 58.1%). Patients having higher education levels were also accompanied by significantly lower prevalence of PIMs (no formal education vs. primary vs. secondary vs. tertiary, 96% vs. 87.3% vs. 84.5% vs. 79.1%) as well as unplanned hospitalization (no formal education vs. primary vs. secondary vs. tertiary, 64.7% vs. 76.2% vs. 40.3% vs. 46.5%).

### 3.2. Indications Associated with Unplanned Hospitalization

The most common indications among hospitalized patients were: CVS and blood related (52.9%), respiratory (23.1 %), and GIT (9.9%) (Table 2).

### 3.3. Commonly Utilized PIMs Prior to Unplanned Hospitalization

The most commonly prescribed PIMs were; N02BA01: aspirin (*n* = 125, 32.4%), A02BC01: omeprazole (*n* = 91, 23.6%), A10AB02: insulin (*n* = 67, 17.4%), A02BC05: esomeprazole (*n* = 33, 8.5%), C08CA01: amlodipine (*n* = 32, 8.3%) and R06AA02: diphenhydramine (*n* = 23, 5.9%) (Table 3).

### 3.4. Educational Level Associated with Polypharmacy, PIMs and Unplanned Hospitalization

Logistic regression analysis evaluated the association of polypharmacy, PIMs and unplanned hospitalization with the educational level of study participants. Results revealed that older patients with no formal education and primary education level were 1.202 times (OR = 1.202, 95% CI = 1.032–2.146, *p*-value = 0.003) and 1.175 times (OR = 1.175, 95% CI = 1.014–1.538, *p*-value = 0.039) as likely to be prescribed polypharmacy as compared to those had tertiary level of education (Table 4). While, examining the association between education level and PIMs, individuals with no formal education were 1.898 times (OR = 1.898, 95% CI = 1.151–2.786, *p*-value = 0.007), respectively, as likely to be prescribed PIMs as compared to those had highest level of education (Table 4). Furthermore, this analysis examined the association between education level and unplanned hospitalization. Results revealed that older patients with no formal education and primary education level were 1.402 times (OR = 1.402, 95% CI = 1.123–1.994, *p*-value = 0.010) and 1.775 times OR = 1.775, 95% CI = 1.281–3.018, *p*-value = <0.001), respectively, as likely to undergo unplanned hospitalization as compared to those had tertiary level of education (Table 4).

## 4. Discussion

The current study set out to determine the influence of patient’s education on the utilization of polypharmacy, PIMs and unplanned hospitalization. The rational for doing so is to inform the healthcare policy makers about the current situation who may take adequate steps for formulating appropriate strategies to prevent this population from untoward effects of improper use of medicines. The findings of present study revealed that prior to unplanned hospitalization most of the patients were prescribed with PIMs including aspirin, omeprazole, insulin, esomeprazole, amlodipine and diphenhydramine, and had been suffering from disorders of CVS, respiratory system, endocrine system and metabolic system.

These results are in line with the previously published study in Nepal where the utilization of PIMs by older patients was quite high and most of them were prescribed with amlodipine (23.16%) and diphenhydramine (4.6%) [44]. Insulin is a frequently used therapeutic agent in the management of diabetes mellitus. But it is also associated with untoward health outcomes like hypoglycemia and seizures. Due to this reason, a national survey in the USA described the use of insulin among 206 older patients per 100,000 outpatient prescription visits as one of the major reasons for unplanned hospitalization [45]. Another study conducted in Karachi, Pakistan had revealed that at least 1 PIM was prescribed to more than half (64%) of the older population [46]. It is suggested that many of the PIMs like antihistaminic agents (e.g., diphenhydramine), proton pump inhibitors (e.g., omeprazole and esomeprazole) and analgesics (e.g., aspirin and non-steroidal anti-inflammatory drugs (NSAIDS)) are more frequently used by older people than any other age group [47,48,49]. This might be attributable to the reason that old age people mostly suffer from insomnia, heart burn, acid reflex, headache, muscle pain, and joint pain due to multiple co-morbidities and the physicians in developing countries like Pakistan are usually inefficient in diagnosing the underlying cause [50,51]. Furthermore, no clinical guidelines have been approved in Pakistan for the diagnosis and management of ailments especially among older patients. Although many international guidelines like Beers criteria [41] and STOP/START criteria [27] are available for assisting in the selection of appropriate medications for this high risk population but unfortunately these guidelines are poorly implemented in public and private healthcare settings of this region [52]. It is the result of urbanization that patient load in public hospitals of Pakistan is increasing day by day. Thus, patients have to wait in long queues for the consultation with physicians. Due to limited hospital timings many patients fail to get consultation from the physicians. In such scenario, physicians give less than 2.5 min per patient and this time duration is inadequate for medical examination and proper counseling of patients [53,54,55]. These are the reasons that account for non-implementation of international guidelines and higher number of PIMs prescribing among older patients.

Logistic regression analysis was used to estimate association of polypharmacy, PIMs, and unplanned hospitalization with participant’s education level. Findings suggested that older patients having no formal and primary level of education were significantly associated with polypharmacy. Similar results were obtained in a national survey conducted on Malaysian older population where patients having primary education were more likely to be prescribed with polypharmacy [56]. A register based survey conducted in Sweden had revealed that older people having low literacy level were significantly associated with the concurrent use of more than five medicines [57]. It is due to the fact that a vast majority of patients having no formal or primary level of education are unaware of their basic health need and expect physicians to prescribe more number of medications [58]. It was also found that older patients with no formal education had significant correlation with the prescribing of PIMs. This finding is parallel with the results of a study conducted in Brazil where similar association between the patient’s education level and the prescribing practices of PIMs was found [59]. Studies revealed that low literacy level is a significant patient related barrier in establishing good relationship between the patients and physicians [60,61,62]. Due to this reason, patients feel hesitation in discussing their health related problems with the physicians. It may have a negative effect on physician’s ability in making the accurate clinical judgment about patient’s health. Thus, such patients may be prescribed with PIMs [63]. Furthermore, like the previously published studies [64,65,66], older people with no formal and primary level of education were found to be significantly associated with unplanned hospitalization. It is primarily because of the fact that such patients were not able to understand and follow the instructions of their healthcare providers properly. Patients having low level of education are less likely to have self-care maintenance and management skills which may have a negative impact on their QoL, thus these patients are at high risk of undergoing unplanned hospitalization as compared to highly educated older patients.

Thus, the rational use of medicines among older patients requires their limited access towards PIMs. A multidisciplinary collaborative approach is needed for defining protocols pertaining to disease management and improving QoL of older patients. As the negligence towards PIMs associated health crises and unplanned hospitalization can economically burden the society and the healthcare system, so it becomes mandatory for policy makers to formulate national action plan and healthcare professionals must implement international treatment guidelines in their routine practice. Also, healthcare professionals must provide proper counseling to all patients especially those having low literacy level. Such patients must be encouraged to communicate openly about their health issues with the physicians and the pharmacists. The health ministry in collaboration with multidisciplinary healthcare providers, national and international organizations should organize educational seminars and campaigns in order to spread awareness regarding health literacy among the local masses especially older patients who have low level of education.

### 4.1. Implication for Policy and Practice

The findings of present study have implications for clinical practice. As there is no literature available that gives an insight about the utilization of healthcare resources and the influence of patient’s education level on polypharmacy, PIMs and unplanned hospitalization in low-middle income countries (LMICs) like Pakistan, so this study plays a substantial role in this context. This study raises the question on the adequacy of clinical practice in Pakistan. The utilization pattern of PIMs reflects unavailability of national guidelines and the disobedience of international guidelines in the management of health related issues among older patients. Such circumstances have threatened the QoL of older patients especially those having low level of education. The promotion of health literacy is mandatory to address these issues.

### 4.2. Strengths and Limitations

To our best knowledge this is a first study in Pakistan that gives an insight about the influence of education level of older people on utilization of PIMs, polypharmacy and unplanned hospitalization. The previously published studies conducted in this region had also considered older people as a high-risk population for irrational use of medicines but the spectrum of their findings was confined to the assessment of utilization pattern of PIMs either during or after hospitalization. This study highlights the need of developing standard treatment protocols and implementing systematic drug monitoring system.

The present study has some limitations. First, this is a cross-sectional study with small sample size, lack of matched control design and data were collected for short duration of time, so the association may not be valid in other populations and countries. Second, the defined daily doses (DDD) of PIMs could not be calculated, so the assumed average maintenance dose per day for a drug used for its main indication was not evaluated. Third, as the appropriateness was checked only for drug indications and interactions, while appropriateness with respect to directions of taking medicines, duplication and duration of therapy was not taken into account, so the investigators were unable to evaluate that either the hospitalization was the result of inappropriate use of PIMs or just having a report of using a PIM prior to admission. In addition, in Pakistan, there are no criteria for assessing PIMs among older patients. Beer’s criteria are not country specific and were specifically designed for older patients of the USA. Medications may be available in Pakistan that are potentially inappropriate but are not included in the list. Therefore, the investigators were unable to evaluate PIMs other than those listed in Beer’s criteria.

## 5. Conclusions

The present study concluded that PIMs were commonly utilized among older patients. The consumption of aspirin, omeprazole, insulin and diphenhydramine was associated with the unplanned hospitalization of these patients. This might be attributable to the unavailability of national standard treatment protocols and disobedience of international guidelines for older patients in Pakistan. The older patients having low level of education were significantly associated with the prescribing practices of PIMs, polypharmacy and unplanned hospitalization as compared to those who had higher level of education. It might be the result of communication gap between prescribers and patients and poor self-care skills. Hence, health literacy must be promoted among older patients especially those having low level of education and provider education regarding PIMS is also needed.

## Figures and Tables

**Table 1:** Characteristics of the participants.

Variables		Education Level				
		No Formal Education (*N* = 150)	Primary (*N* = 63)	Secondary (*N* = 129)	Tertiary (*N* = 43)	Total (*N* = 385)
		*n* (%)	*n* (%)	*n* (%)	*n* (%)	*n* (%)
Gender	Male	77 (51.3)	47 (74.6)	98 (75.9)	33 (76.7)	255 (66.2)
	Female	73 (48.7)	16 (25.4)	31 (24.0)	10 (23.3)	130 (33.8)
Age	65–74	99 (66.0)	39 (61.9)	97 (75.1)	27 (62.8)	262 (68.1)
	75–84	39 (26.0)	21 (33.3)	28 (21.7)	13 (30.2)	101 (26.2)
	≥85	12 (8.0)	3 (4.8)	4 (3.1)	3 (6.9)	22 (5.7)
Civil Status	Single	3 (2.0)	4 (6.4)	2 (1.6)	1 (2.3)	10 (2.6)
	Married	116 (77.3)	55 (87.3)	108 (83.7)	33 (76.7)	312 (81.0)
	Widow	29 (19.3)	4 (6.4)	14 (10.9)	8 (18.6)	55 (14.3)
	Divorced	2 (1.3)	0 (0.0)	5 (3.9)	1 (2.3)	8 (2.1)
Employment Status	Employed	37 (24.7)	34 (53.9)	98 (75.9)	39 (90.7)	208 (54.0)
	Unemployed	113 (75.3)	29 (46.0)	31 (24.0)	4 (9.3)	177 (45.9)
Annual Income	Low class	140 (93.3)	55 (87.3)	89 (68.9)	5 (11.6)	289 (75.1)
	Middle class	9 (6.0)	8 (12.7)	31 (24.0)	15 (34.9)	63 (16.4)
	Upper class	1 (0.7)	0 (0.0)	9 (6.9)	23 (53.5)	33 (8.6)
Residence	Rural	84 (56.0)	23 (36.5)	22 (17.1)	2 (4.7)	131 (34.0)
	Urban	66 (44.0)	40 (63.5)	107 (82.9)	41 (95.4)	254 (65.9)
Self- reported Health	Good	8 (5.3)	8 (12.7)	10 (7.8)	3 (6.9)	29 (7.5)
	Moderate	80 (53.3)	36 (57.1)	92 (71.3)	23 (53.5)	231 (60.0)
	Poor	62 (41.3)	19 (30.2)	27 (20.9)	17 (39.5)	125 (32.5)
Health service utilization	Number of Clinic Visits of ≤3/year	75 (50.0)	30 (47.6)	54 (41.9)	13 (30.2)	172 (44.7)
	Number of Clinic Visits of ≥4/year	75 (50.0)	33 (52.4)	75 (58.1)	30 (69.8)	213 (55.3)
Health Risks	Smoking	61 (40.7)	28 (44.4)	65 (50.4)	18 (41.9)	172 (44.7)
	Alcohol Consumption	0 (0.0)	0 (0.0)	0 (0.0)	1 (2.3)	1 (0.3)
	Obesity	34 (22.7)	14 (22.2)	36 (27.9)	10 (23.3)	94 (24.4)
	None	55 (36.7)	21 (33.3)	28 (21.7)	14 (32.6)	118 (30.7)
Co-morbidity	Present	111 (74.0)	44 (69.8)	104 (80.6)	36 (83.7)	295 (76.6)
	Absent	39 (26.0)	19 (30.2)	25 (19.4)	7 (16.3)	90 (23.4)
Number of Drugs	Four or less	39 (26.0)	19 (30.2)	51 (39.5)	18 (41.9)	127 (32.9)
	Polypharmacy (five to nine)	103 (68.7)	39 (61.9)	70 (54.3)	22 (51.2)	234 (60.8)
	Excessive Polypharmacy (ten or more)	8 (5.3)	5 (7.9)	8 (6.2)	3 (6.9)	24 (6.2)
PIMS	Yes	144 (96.0)	55 (87.3)	109 (84.5)	34 (79.1))	342 (88.8)
	No	6 (4.0)	8 (12.7)	20 (15.5)	9 (20.9)	43 (11.2)
Unplanned hospitalization	Yes	97 (64.7)	48 (76.2)	52 (40.3)	20 (46.5)	217 (56.4)
	No	53 (35.3)	15 (23.8)	77 (59.7)	23 (53.5)	168 (43.6)

**Table 2:** Indications associated with older hospitalized patients.

Sr.	Indications		Education Level				
			No Formal Education (*N* = 150)	Primary (*N* = 63)	Secondary (*N* = 129)	Tertiary (*N* = 43)	Total (*N* = 385)
			*n* (%)	*n* (%)	*n* (%)	*n* (%)	*n* (%)
1	CVS & Blood related	Ischemic heart diseases	18 (12.0)	9 (14.3)	14 (10.9)	11 (25.6)	52 (13.5)
		Arrhythmias	34 (22.7)	13 (20.6)	32 (24.8)	15 (34.9)	94 (24.4)
		Angina pectoris	23 (15.3)	6 (9.5)	27 (20.9)	2 (4.7)	58 (15.1)
	Total		75 (50.0)	28 (44.4)	73 (56.6)	28 (65.1)	204 (52.9)
2	CNS	Insomnia	6 (4.0)	3 (4.8)	4 (3.1)	2 (4.7)	15 (3.9)
		Anxiety	1 (0.7)	1 (1.6)	7 (5.4)	2 (4.7)	11 (2.9)
	Total		7 (4.7)	4 (6.4)	11 (8.5)	4 (9.3)	26 (6.8)
3	GIT	Dyspepsia	11 (7.3)	4 (6.4)	3 (2.3)	2 (4.7)	20 (5.2)
		Anorexia	7 (4.7)	3 (4.8)	2 (1.6)	0 (0.0)	12 (3.1)
		Gastritis	2 (1.3)	1 (1.6)	3 (2.3)	0 (0.0)	6 (1.6)
	Total		20 (13.3)	8 (12.7)	8 (6.2)	2 (4.7)	38 (9.9)
4	Respiratory	Pneumonitis	6 (4.0)	9 (14.3)	5 (3.9)	0 (0.0)	20 (5.2)
		Dyspnea	21 (14.0)	6 (9.5)	13 (10.1)	4 (9.3)	44 (11.4)
		Pulmonary edema	4 (2.7)	7 (11.1)	2 (1.6)	0 (0.0)	13 (3.4)
		Pulmonary fibrosis	7 (4.7)	0 (0.0)	4 (3.1)	1 (2.3)	12 (3.1)
	Total		38 (25.3)	22 (34.9)	24 (18.6)	5 (11.6)	89 (23.1)
5	Kidney	Urinary tract infections	1 (0.7)	0 (0.0)	3 (2.3)	0 (0.0)	4 (1.0)
		Nephritis	0 (0.0)	0 (0.0)	1 (0.8)	1 (2.3)	2 (0.5)
	Total		1 (0.7)	0 (0.0)	4 (3.1)	1 (2.3)	6 (1.6)
6	Joint & Muscles related	Fractures	2 (1.3)	1 (1.6)	5 (3.9)	0 (0.0)	8 (2.1)
		Arthritis	0 (0.0)	0 (0.0)	1 (0.8)	0 (0.0)	1 (0.3)
	Total		2 (1.3)	1 (1.6)	6 (4.7)	0 (0.0)	9 (2.3)
7	Endocrinology & metabolic disorders	Diabetes mellitus	2 (1.3)	0 (0.0)	1 (0.8)	0 (0.0)	3 (0.8)
		Thyroid disorders	2 (1.3)	0 (0.0)	0 (0.0)	0 (0.0)	2 (0.5)
	Total		4 (2.7)	0 (0.0)	1 (0.8)	0 (0.0)	5 (1.3)
8	Others		3 (2.0)	0 (0.0)	2 (1.6)	3 (6.9)	8 (2.1)

**Table 3:** Potentially inappropriate medications (PIMs) prescribing pattern among study participants.

Sr.	Medicines	ATC Code	No Formal Education (*N* = 150)	Primary (*n* = 63)	Secondary (*N* = 129)	Tertiary (*N* = 43)	Total (*n* = 385)	Trend
1	Aspirin	N02BA01	56 (37.3)	28 (44.4)	30 (23.3)	11 (25.6)	125 (32.4))	High
2	Amlodipine	C08CA01	18 (12.0)	9 (14.3)	4 (3.1)	1 (2.3)	32 (8.3)	Medium
3	Alprazolam	N05BA12	5 (3.3)	6 (9.5)	4 (3.1)	0 (0.0)	15 (3.8)	Low
4	Atropine	A03BA01	8 (5.3)	3 (4.8)	0 (0.0)	3 (7.0)	14 (3.6)	Low
5	Aripiprazole	N05AX12	1 (0.7)	0 (0.0)	0 (0.0)	0 (0.0)	1 (0.2)	Low
6	Chlorpheniramine	R06AB04	1 (0.7)	1 (1.6)	2 (1.6)	0 (0.0)	4 (1.0)	Low
7	Clonazepam	N03AE01	1 (0.7)	2 (3.2)	0 (0.0)	3 (7.0)	6 (1.5)	Low
8	Chlorzoxazone	M03BB03	2 (1.3)	0 (0.0)	0 (0.0)	0 (0.0)	2 (0.5)	Low
9	Caffeine	N06BC01	1 (0.7)	0 (0.0)	0 (0.0)	0 (0.0)	1 (0.2)	Low
10	Chlorpromazine	N05AA01	0 (0.0)	2 (3.2)	0 (0.0)	0 (0.0)	2 (0.5)	Low
11	Diclofenac Sodium	M01AB05	9 (6.0)	4 (6.3)	9 (7.0)	0 (0.0)	22 (5.7)	Medium
12	Dimenhydrinate	R06AA52	4 (2.7)	2 (3.2)	2 (1.6)	4 (9.3)	12 (3.1)	Low
13	Digoxin	C01AA05	5 (3.3)	3 (4.8)	0 (0.0)	0 (0.0)	8 (2.0)	Low
14	Diphenhydramine	R06AA02	7 (5.3)	2 (3.2)	9 (7.0)	5 (11.6)	23 (5.9)	Medium
15	Diltiazem HCL	C08DB01	4 (2.7)	1 (1.6)	0 (0.0)	0 (0.0)	5 (1.2)	Low
16	Dexamethasone	R01AD03	3 (2.0)	5 (7.9)	0 (0.0)	3 (7.0)	11 (2.8)	Low
17	Doxazosin	C02CA04	0 (0.0)	1 (1.6)	0 (0.0)	0 (0.0)	1 (0.2)	Low
18	Ephedrine	R01AA03	2 (1.3)	0 (0.0)	0 (0.0)	0 (0.0)	2 (0.5)	Low
19	Esomeprazole	A02BC05	16 (10.7)	3 (4.8)	9 (7.0)	5 (11.6)	33 (8.5)	Medium
20	Famotidine	A02BA03	1 (0.7)	0 (0.0)	0 (0.0)	0 (0.0)	1 (0.2)	Low
21	Flurbiprofen	R02AX01	1 (0.7)	0 (0.0)	0 (0.0)	0 (0.0)	1 (0.2)	Low
22	Glimepiride	A10BB12	3 (2.0)	2 (3.2)	4 (3.1)	0 (0.0)	9 (2.3)	Low
23	Glibenclamide	A10BB01	7 (5.3)	0 (0.0)	0 (0.0)	0 (0.0)	7 (1.8)	Low
24	Hydrocortisone	D07AA02	9 (6.0)	4 (6.3)	5 (3.9)	4 (9.3)	22 (5.7)	Low
25	Insulin	A10AB02	20 (13.3)	13 (20.6)	23 (17.8)	11 (25.6)	67 (17.4)	High
26	Ibuprofen	M01AE01	4 (2.7)	0 (0.0)	0 (0.0)	0 (0.0)	4 (1.0)	Low
27	Ketorolac	M01AB15	1 (0.7)	2 (3.2)	0 (0.0)	0 (0.0)	3 (0.7)	Low
28	Methylprednisolone	D07AC14	2 (1.3)	1 (1.6)	0 (0.0)	0 (0.0)	3 (0.7)	Low
29	Metoclopramide	A03FA01	5 (3.3)	2 (3.2)	0 (0.0)	0 (0.0)	7 (1.8)	Low
30	Meloxicam	M01AC06	0 (0.0)	1 (1.6)	0 (0.0)	0 (0.0)	1 (0.2)	Low
31	Nifedipine	C08CA05	7 (5.3)	1 (1.6)	0 (0.0)	0 (0.0)	8 (2.0)	Low
32	Naproxen	M02AA12	3 (2.0)	3 (4.8)	0 (0.0)	0 (0.0)	6 (1.5)	Low
33	Omeprazole	A02BC01	30 (20.0)	18 (28.6)	28 (21.7)	15 (34.9)	91 (23.6)	High
34	Orphenadrine	N04AB02	9 (6.0)	6 (9.5)	0 (0.0)	7 (16.3)	16 (4.1)	Low
35	Piroxicam	M01AC01	4 (2.7)	4 (6.3)	0 (0.0)	2 (4.7)	10 (2.5)	Low
36	Promethazine	R06AD02	1 (0.7)	0 (0.0)	0 (0.0)	0 (0.0)	1 (0.2)	Low
37	Prednisolone	H02AB06	5 (3.3)	4 (6.3)	6 (4.7)	3 (7.0)	18 (4.6)	Low
38	Paroxetine	N06AB05	3 (2.0)	1 (1.6)	0 (0.0)	0 (0.0)	4 (1.0)	Low
39	Pantoprazole	A02BC02	1 (0.7)	2 (3.2)	0 (0.0)	0 (0.0)	3 (0.7)	Low
40	Ranitidine	A02BA02	5 (3.3)	7 (11.1)	5 (3.9)	2 (4.7)	19 (4.9)	Low
41	Rabeprazole	A02BC04	4 (2.7)	2 (3.2)	0 (0.0)	0 (0.0)	6 (1.5)	Low
42	Resperidone	N06AX08	1 (0.7)	1 (1.6)	0 (0.0)	0 (0.0)	2 (0.5)	Low
43	Tramadol	N02AX02	6 (4.0)	2 (3.2)	6 (4.7)	4 (9.3)	18 (4.6)	Low
44	Theophylline	R03DA04	5 (3.3)	2 (3.2)	3 (2.3)	0 (0.0)	10 (2.5)	Low

**Table 4:** Logistic regression analysis of educational level associated with polypharmacy, PIMs, and unplanned hospitalization.

Independent Variable		Dependent Variable		OR	95% CI	*p*-Value
		Yes	No			
**A: Polypharmacy**						
Education Level	No formal education	111 (28.8)	39 (10.1)	1.202	1.032–2.146	0.003
	Primary	44 (11.4)	19 (4.9)	1.175	1.014–1.538	0.039
	Secondary	78 (20.3)	51 (13.2)	1.038	0.522–1.567	0.067
	Tertiary	25 (6.5)	18 (4.7)	1.0	-----	-----
**B: PIMs**						
Education Level	No formal education	144 (37.4)	6 (1.6)	1.898	1.151–2.786	0.007
	Primary	55 (14.3)	8 (2.1)	1.232	0.642–1.815	0.072
	Secondary	109 (28.3)	20 (5.2)	1.107	0.953–1.760	0.058
	Tertiary	34 (8.8)	9 (2.3)	1.0	-----	-----
**C: Unplanned hospitalization**						
Education Level	No formal education	97 (25.2)	53 (13.8)	1.402	1.123–1.994	0.010
	Primary	48 (12.5)	15 (3.9)	1.775	1.281–3.018	<0.001
	Secondary	52 (13.5)	77 (20.0)	0.438	0.122–1.177	0.207
	Tertiary	20 (5.2)	23 (6.0)	1.0	-----	-----
